# Next Generation Sequencing Technologies for Insect Virus Discovery

**DOI:** 10.3390/v3101849

**Published:** 2011-10-10

**Authors:** Sijun Liu, Diveena Vijayendran, Bryony C. Bonning

**Affiliations:** Department of Entomology, Iowa State University, Ames, IA 50011, USA; E-Mails: sliu@iastate.edu (S.L.); diveena@iastate.edu (D.V.)

**Keywords:** insect virus, next generation sequencing, virus discovery, small RNA, transcriptome

## Abstract

Insects are commonly infected with multiple viruses including those that cause sublethal, asymptomatic, and latent infections. Traditional methods for virus isolation typically lack the sensitivity required for detection of such viruses that are present at low abundance. In this respect, next generation sequencing technologies have revolutionized methods for the discovery and identification of new viruses from insects. Here we review both traditional and modern methods for virus discovery, and outline analysis of transcriptome and small RNA data for identification of viral sequences. We will introduce methods for *de novo* assembly of viral sequences, identification of potential viral sequences from BLAST data, and bioinformatics for generating full-length or near full-length viral genome sequences. We will also discuss implications of the ubiquity of viruses in insects and in insect cell lines. All of the methods described in this article can also apply to the discovery of viruses in other organisms.

## 1. Introduction

Viruses can be found wherever life is present and are likely to be the most abundant and diverse biological entities on earth [1–3]. In addition to increased understanding of their diversity and evolution, the viruses associated with insects are of particular interest from the standpoints of (a) protection of beneficial insects from virus infection (e.g., the honey bee, *Apis mellifera* L. [4]; silk moths, *Bombyx mori* L.), (b) practical use of insect viruses for management of pestiferous insects including invasive species, (e.g., various lepidopteran pests including the codling moth, *Cydia pomonella* Linnaeus and the velvet bean caterpillar, *Anticarsia gemmatalis* (Hübner) [5], (c) identification of insects that vector viruses important to human, animal and plant health [6], and (d) use of insect viruses as vectors for protein expression or gene silencing, and adaptation of virus-like particles for a variety of purposes [7,8]. Contrary to the typical view of viruses as pathogens, viruses may also have mutualistic or symbiotic relationships with their hosts, which are of fundamental interest [9]. For example, polydnaviruses are required for the survival of parasitoid wasps as they develop in the host insect [10]. A densovirus has been reported to function in wing morph determination of the host aphid [11]. A bacteriophage that infects the aphid facultative endosymbiont, *Hamiltonella defensa*, protects the pea aphid from attack by the parasitoid *Aphidius ervi* by killing the developing wasp larva [12,13].

Traditionally, viruses were isolated from insects that displayed an abnormal phenotype as a result of virus infection. While infection with some insect viruses, such as baculoviruses, results in clear symptoms and ultimately death of the host, many virus infections are asymptomatic. In recent years, with the development of the Next Generation Sequencing (NGS) technologies, it has become evident that asymptomatic or covert virus infections are ubiquitous. These viruses may accumulate to relative low titers in the host organism (*i.e.*, in a chronic infection), or become latent, such that virus production ceases altogether. These viruses would not readily be detected by use of traditional protocols for virus discovery.

The use of NGS over the past five years has revolutionized the discovery of microorganisms and viruses. The technology allows for rapid, inexpensive, high throughput and accurate sequencing for identification of viral sequences derived from whole insects or specific tissues, and for viruses present at low titers that do not cause symptoms in the host. NGS has also been used for virus surveillance, for arthropod-borne viruses for example [14].

## 2. Conventional Approaches to Virus Discovery

The first virus discovered was Tobacco mosaic virus (TMV) discovered by Dmitri Iwanowsk, a Russian botanist in 1882. He showed that extracts from diseased tobacco plants could transmit disease to other plants after passage through ceramic filters that were sufficiently fine to retain bacteria. The first virus particles (TMV) were observed following the invention of the electron microscope in 1931. Although it was known from the 1930s that viruses consisted of a protein shell and nucleic acids, methods for detection of viral protein and viral genomic RNA or DNA were not developed until the late 1970s and early 1980s. Conventional approaches for virus discovery are to collect insects from multiple populations from multiple locations, and use several micrograms of material at minimum for virus purification. Virus purification protocols vary, but material is typically homogenized, centrifuged and filtered under sterile conditions. Further purification may include ultracentrifugation and sucrose or cesium chloride gradient steps. The sample may then be used for visualization of virus particles by electron microscopy, infection of cultured insect cells and observation for cytopathic effects [15–17], and infection of insects by spraying, injection or oral inoculation to fulfill Koch’s postulates. Viruses would then be identified and further characterized by use of serological methods and nucleic acid hybridization (where specific antisera or probes are available), molecular cloning and genomic sequencing [18]. Use of a cell line is beneficial in that it allows for culture and amplification of viruses that cause either acute or covert infections in the host. However, the lack of appropriate insect cell lines for such virus screens is a common limiting factor and it cannot be assumed that all viruses present in an insect would replicate in a given cell line.

### 2.1. Electron Microscopy

**Figure 1.**
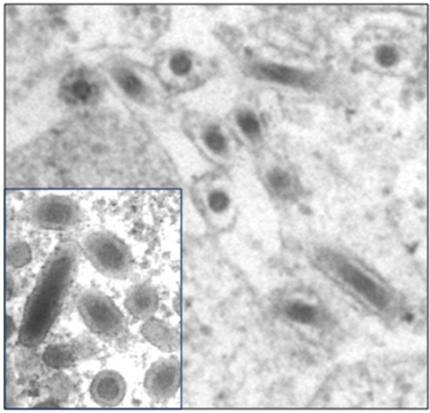
Transmission electron micrographs of the enveloped nucleocapsids of a baculovirus (Autographa californica nucleopolyhedrovirus; Baculoviridae). Inset: virions of White spot syndrome virus (WSSV; Whispoviridae) of shrimp. Based on morphology, WSSV was initially thought to be a baculovirus. TEM courtesy of Hailin Tang.

Electron microscopy (EM) has been and continues to be one of the most important techniques for virus discovery [19]. Indeed early classification of viruses depended heavily on the morphology of the viral capsid as revealed by EM. One of the primary advantages for the use of EM is that organism or virus specific reagents are not required for virus identification. Although identification of a virus beyond the family level may not be possible, EM provides leads for more detailed characterization of the virus. In addition EM provides important confirmation of the presence of a virus following detection of viral sequences by molecular means. An additional advantage for the use of EM is that samples stored under conditions that would not allow for molecular testing or virus culture can be used for rapid EM visualization of viruses. There are numerous examples of insect-derived viruses depicted by EM in the published literature for which no further characterization has been undertaken [20]. Subsequent studies on characterization of viruses from the same host insect commonly fail to refer back to the electron micrographs in older papers [21,22]. In some instances, initial classification based on morphology was subsequently revised based on molecular information. This was the case for White spot syndrome virus of shrimp, which was initially believed to be a baculovirus [23] (Figure 1).

Samples used for EM analysis for virus observation range from crudely extracted samples to viruses purified via ultracentrifugation and sucrose or cesium chloride gradients. Thin sectioning of tissues is also common for observing the tissue tropism of insect viruses. For examination of virus particles, specimens are placed on to grids and typically subjected to negative staining [24]. Commonly used negative stains are 0.05 to 2% uranyl acetate, 1 to 2% phosphotungstic acid (PTA), and 0.05 to 5% ammonium molybdate. Immuno-electron microscopy (IEM) [25], which uses antibody-virus reactions for virus detection by EM was first developed in 1941 [26]. For IEM, viruses in solution are mixed with the viral antiserum to form a virus-antibody complex or immunoaggregate. This antibody coated virus particle can be negatively stained and distinguished by EM.

Immunolocalization is used for observing virus in thin sectioned tissue specimens and for specifically identifying known viruses. The viruses are coated with viral antibodies followed by secondary antibody conjugated with colloidal gold (gold labeling) in the grid. The grid is then negatively stained.

### 2.2. Serological Methods

Two serological methods that are commonly used for virus detection are enzyme-linked immunosorbant assay (ELISA) and western blot. Both methods employ antibodies that recognize viral coat or other viral proteins to detect the presence of a given virus either from samples of purified virus or from total proteins extracted from infected insects or tissues. ELISA and western blot are widely used for detection of known viruses and may also be used to assess the serological relationship of a new virus to known viruses within the same family.

### 2.3. Standard Molecular Methods

Nucleic acid hybridization (Southern blot, northern blot and dot blot) are also useful for identification of viruses, but these methods also rely on prior knowledge of the target virus; Viral specific nucleic acid probes are labeled for hybridization to the target viral DNA or RNA to demonstrate the presence of the virus.

Polymerase chain reaction (PCR) and reverse transcription polymerase chain reaction (RT-PCR) are used to amplify viral DNA or RNA respectively, either full length genomic sequences, or parts thereof. The resulting DNA or cDNA fragments are cloned into vectors and used for sequencing, and in the case of full length sequences may also be used for screening for infectious virus clones [27]. Comparison of sequence data for a given virus to those of known viruses will indicate whether the virus is novel or similar to known viruses, and facilitates virus classification and phylogenetic analyses.

### 2.4. EST Libraries

Several insect viruses were discovered through analysis of expressed sequence tag (EST) libraries. EST libraries are produced by isolating total RNA from insects, purifying mRNA, and generating and sequencing a cDNA library. Hence ESTs are sequences (typically 500–800 nt) that represent the sequences of transcribed genes. Purification of mRNA to generate the cDNA library requires selection of RNAs that include polyA tails on a polyT column, thereby limiting sequence representation to viral RNAs with polyA tails. Although relatively little viral sequence with low coverage is provided by ESTs, there may be sufficient sequence for 5′ RACE to acquire the full genomic sequence. Given the low sequence coverage provided by EST libraries, virus detection via EST sequences is likely to be limited to viruses that are present at high titers in the host insect.

Valles *et al.* [28] detected six ESTs of putative viral origin in a cDNA library derived from the red imported fire ant, *Solenopsis invicta*, which causes significant economic damage in the U.S. Three of these ESTs exhibited significant homology to Acute bee paralysis virus (Dicistroviridae) and 5′ RACE was used to delineate the entire genome sequence of the virus, Solenopsis invicta virus 1 (SINV-1).

Hunnicutt *et al.* [29] isolated Homalodisca coagulata virus-1 (HoCV-1) following detection of viral sequences in EST libraries from the glassy-winged sharpshooter, *H. vitripennis* (also known as *H. coagulata*) [30]. This insect was introduced from the southeastern U.S. to California in the late 1980s and wreaked havoc as a result of its polyphagy and in the absence of natural enemies such as parasitic wasps and entomopathogenic fungi. In addition, *H. vitripennis* vectors the bacterium *Xylella fastidiosa*, which negatively impacts numerous plant species. Viruses isolated from this insect may have potential for use in its management. Sequences derived from a phytoreovirus (plant virus) were also detected from an *H. vitripennis* salivary gland cDNA library [31].

Oliveria *et al.* [32] discovered three novel small RNA viruses (NvitV-1, -2 and -3) with the longest contig (*i.e.*, series of overlapping DNA sequences used to reconstruct the original sequence) of 2789 nt (NvitV-1; not including the polyA) from the parasitoid wasp, *Nasonia vitripennis*, by data mining of EST libraries.

### 2.5. Microarrays

The use of microarrays was proposed and tested for detection and genotyping of pathogens [33]. The DNA microarray-based platform was designed to include all viruses that had full-length sequences in GenBank and included the most highly conserved 70mer sequences from every fully sequenced reference viral genome. The microarray was used for both genotyping of viruses and for virus discovery. In addition to identifying viruses present in a sample, hybridized viral sequences were isolated from the spot in the microarray, amplified by PCR, cloned and sequenced for identification of novel viruses [34]. The combination of array hybridization followed by direct viral sequence recovery allows for rapid characterization of novel viruses. Microarrays have not been adapted for invertebrate virus discovery, but offer an alternative approach; for example, an Arthropod Pathogen microarray was used for detection (but not discovery) of known viruses in honey bees [35].

## 3. Next Generation Sequencing for Virus Discovery

Next Generation Sequencing is a non-Sanger-based and high-throughput methodology [36] which allows for generation of millions of sequences at once [37]. Multiple high-throughput sequencing technologies have been developed [38–40]. The most common NGS platforms are Roche 454 pyrosequencing (454 Life Science), Illumina (Solexa) sequencing, and SOLiD sequencing (ABI Biosystems) (Table 1).

### 3.1. Sample and Library Preparation

Viral sequences can be extracted from either total DNA (for DNA viruses only) or RNA isolated from insects [41]. Alternatively, prior to viral DNA or RNA extraction, virus purification can be conducted to eliminate host nucleic acid contamination, followed by extraction of viral DNA or RNA [42]. Insects collected from the field should ideally be processed rapidly with RNA stored in RNAlater (Qiagen) or TRIzol (Invitrogen), and DNA stored in DNAzol (Invitrogen) at −80 °C for later processing. However, viral RNA and DNA can also be stored safely in crushed insects in such stabilizing solutions: While the viral RNA and DNA under these conditions are stable at room temperature, it is recommended that samples be kept cold. Alternatively, insects can be stored directly at −80 °C, although some RNA viruses (e.g., some dicistroviruses) are unstable on freeze-thawing.

Methods used for library preparation vary according to the platform used for sequencing. Reagents, kits and methods for preparing libraries can be obtained from the corresponding companies. In general, there are three types of libraries that are most useful in the context of virus discovery: DNA, RNA (including transcriptome) and small RNA libraries. For transcriptome sequencing, mRNA is extracted from total RNA by polyT treatment or by methods for ribosomal RNA depletion [14] before being used for library construction. Procedures for library preparation normally include DNA or RNA fragmentation (DNA and transcriptome sequencing), size selection of fragments, addition of adapters, PCR or RT-PCR (for transcriptome and small RNA libraries), and amplification of sequences. Following library construction, sequencing is carried out.

### 3.2. Bioinformatics Analysis

There are no standard methods for analysis of sequences generated by NGS [43], although numerous bioinformatics methods and pipelines have been developed as dictated by the specific challenges of the datasets generated [44]; for example, data analysis is greatly simplified by the presence of a reference genome against which to align and compare NGS sequences. In general, the initial raw sequencing data (reads) are treated with programs provided by the manufacturers for base calling, removal of adaptor sequences (adaptors are usually a the 5′-end) and removal of low quality reads. For small RNA sequencing, the 3′-end adaptors are trimmed by either customer-developed programs or programs such as Cutadapt [45] which are freely available. Different researchers have used different approaches for data mining to find viral sequences: DNA or transcriptome sequence data can be used to conduct BLAST (**B**asic **L**ocal **A**lignment **S**earch **T**ool) searches (blastx, tblastx, or blastn [46] against NCBI non-redundant (nr) databases, or a viral database [35] before the reads are assembled. The reads that hit viral sequences with given E-values are extracted and used for *de novo* assembly. Many programs for short-read assembly are available [40] and can be used for either *de novo* assembly or mapping the reads to known viral genomes. For small RNA sequencing data, the reads may be assembled *de novo*, and the contigs then used for BLAST analysis to find homologous viral sequences. The contigs with viral sequence hits may be extracted and reassembled for further characterization. The bioinformatics methods used for virus discovery by NGS data mining are summarized in Table 2.

### 3.3. Confirmation of NGS-Derived Viral Sequences

Following detection of viral sequences by NGS technologies, the presence of viral sequences in the sample must be confirmed by PCR (DNA viruses) or RT-PCR (RNA viruses). Real time PCR/RT-PCR can be used to quantify the amount of virus present, and provide validation for the number of observed reads in the NGS datasets. To confirm whether the identified virus replicates in the host insect, RT-PCR for detection of viral transcripts, or negative strand-specific RT-PCR for ssRNA viruses can be performed. The use of tagged primers for enhanced specificity is recommended [47]. Detection of negative strand RNA is used as an indicator of replication for positive strand RNA viruses. Acquisition of additional supporting evidence to confirm the presence of the virus is strongly recommended (e.g., virus purification, EM analysis, detection of viral coat proteins, isolating genomic DNA or RNA, or showing virus increase over time by using quantitative RT-PCR/PCR); On occasion, the sequences of viruses that do not replicate in the target insect, but are present in the diet of that insect [28], or have become incorporated into the host genome are detected. Hence, detection of viral sequences in a particular insect is not sufficient evidence for replication of the virus in that host. Ideally, it would be possible to purify the virus and infect other individuals of the same species that lack the virus (*i.e.*, fulfill Koch’s postulates).

## 4. Discovery of Insect Viruses by NGS

Next Generation Sequencing has been widely applied [38,48–50] including for the discovery of novel microbes and viruses from animals and plants [51,52]. To date, there are about a dozen reports of viruses discovered from insects or insect cell cultures by means of NGS.

### 4.1. DNA and Transcriptome Sequencing

The first application of NGS technology that demonstrated the potential use of this approach for virus discovery was a metagenomic analysis of the honey bee, *Apis mellifera* L., conducted to elucidate the causes of colony collapse disorder (CCD) [41,55]. The pathogens of bees, including more than 18 viruses [56], have been well studied. No new viruses were detected during the course of this analysis. For the metagenomic analysis, total RNA was extracted from bees taken from CCD and non-CCD colonies collected from the US, and Australia and also from royal jelly from China. The RNA libraries were subjected to 454 pyrosequencing, and raw reads were trimmed and assembled into contigs. Contigs were used for BLAST analysis (blastn and blastx) [46] against the NCBI nr database. Seven viruses were identified in bees derived from CCD colonies (Table 3), compared to five from non-CCD colonies. A wide range of other pathogens were also detected [41]. The presence of the viruses was confirmed by RT-PCR and Sanger sequencing, and the presence of Israeli acute paralysis virus (IAPV) was found to be a significant indicator of CCD. Shortly thereafter, IAPV-like viruses were detected in a fresh water lake, in a metagenomic analysis of the viral community in fresh water [57].

Analysis of the microbiome of the honey bee over time was used to identify four novel viruses [35] including two which were the most abundant components of the microbiome at ∼10^11^ viruses per bee. High frequency sampling along with molecular detection methods including a custom arthropod pathogen microarray, qPCR, and deep sequencing were used for episodic viral detection throughout the year. Total nucleic acids from 20 monitor hives (3 μg nucleic acids per hive) were pooled and three sequencing libraries prepared (one DNA, two RNA libraries). The RNA libraries were constructed with various modifications (e.g., with and without purification of mRNA) to optimize the detection of viruses, bacteria, fungi/protists, mites, and nematodes. Sequencing was performed with paired-end 65 nt reads by using an Illumina GAII. To analyze the sequencing data, a database was created that included all of the complete arthropod virus genome sequences available at the time. The entire sequencing dataset was queried against the arthropod virus library by using blastn and tblastx, and hits with a minimum e-value of 1 × 10^−3^ used for further analysis. Hits were assembled using the Geneious sequence analysis package. Contigs (>250 nt) were queried again against the dataset by tblastx with an e-value ≤ 1 × 10^−5^. The positive hits were then queried against the nr database with the same parameters to eliminate spurious hits. For the contigs that appeared divergent or that hit non-honey bee associated viruses, extension of the contigs was performed using the entire read dataset with a paired-end contig extension program. From this analysis, it appeared that overall virus incidence was sporadic, although the use of only five bees per sample from each hive and a virus detection limit of 1.9 × 10^5^ virus genome copies may explain the apparent disappearance of some viruses over time. Four new viruses were discovered from the honey bees, including two dicistroviruses (named Aphid lethal paralysis virus strain Brookings, and Big Sioux River virus), and two viruses for which the complete genome sequence was acquired (Lake Sinai virus 1 and Lake Sinai virus 2; Table 3). Replication of LSV1 and LSV2 in the honey bee was confirmed by RT-PCR.

A metagenomic analysis of coastal RNA viruses also revealed viruses that are distantly related to viruses of arthropods, including dicistroviruses [52,62].

### 4.2. Virus Purification Followed by NGS

A so-called vector-enabled metagenomics (VEM) approach was used to examine the diversity of DNA viruses present in multiple species of mosquitoes from California, U.S. [42]. In this approach, viruses were purified from mosquito samples by homogenization of the samples, filtration and a cesium chloride step gradient. The presence of viral particles and the absence of bacterial and eukaryotic cells were confirmed prior to further processing. Total DNA was extracted and amplified prior to 454 sequencing on a GS20 or GS FLX pyrosequencing platform. Short reads were removed from the sequencing dataset prior to blastn and tblastx analysis against the GenBank nr database for identification of viral sequences and further assembly and annotation. The presence of some of the viral sequences in the mosquitoes was confirmed by PCR. Remarkably, the sequences of 107 DNA viruses derived from 16 viral families were identified in the three mosquito samples. Viruses detected included viruses of animals, plants, insects and bacteria with the majority being densoviruses. Although novel viruses were detected, few full length genome sequences were acquired. The pooling of multiple species of mosquito also prevented immediate identification of the host species of novel viruses.

The first insect nidovirus, Cavally virus (CAVV), was discovered following virus isolation from mosquito heads, and amplification in the C6/36 mosquito cell line. The virus was titrated on insect cells and cell culture supernatant used as a source of pure virus [17]. Virions were visualized by TEM and RNA extracted from purified virus for high through-put and conventional sequencing. The ssRNA genome is 20 kb in size. CAVV was present in 9% of mosquitoes sampled around the primary forest habitat in Ivory Coast, and virus incidence increased with increasing human habitation.

### 4.3. Sequencing of Small RNA

**Figure 2.**
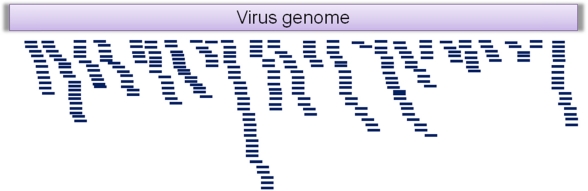
Alignment of short interfering RNAs (siRNA) derived from viral RNA can be used to delineate viral genomic sequences.

RNA interference (RNAi) plays a vital role in defense against RNA viruses in a wide range of organisms including insects [63–72]. The enzyme Dicer recognizes double stranded (ds) RNA (produced during the replication of RNA viruses) and cleaves it into small interfering RNAs (siRNAs) of about 22 nt in length [73]. Argonaute, a protein component of the RNA-induced silencing complex (RISC), binds the antisense strand of the siRNA and degrades viral RNA complementary to the siRNA [74]. Hence, sequencing of small RNAs (sRNA: 17–30 nt) and assembly of the virus-derived siRNAs can be used to reveal the sequences of RNA viruses present in an insect (Figure 2).

The first report of the use of sRNA sequencing for virus identification was for analysis of the sweet potato [75]. In this case, the authors inoculated the plants with known RNA viruses, Sweet potato feathery mottle potyvirus (SPFMV) and Sweet potato chlorotic stunt closterovirus (SPCSV). Small RNA was isolated from the inoculated plants and sequenced by using Illumina GAII. The sRNA reads were assembled with three different assembly programs for sequence assembly, and contigs were reassembled to generate longer contigs using the program Contigexpress (Vector NTI, Invitrogen). The contigs were queried by searching the GenBank nr database for viral sequences. SPFMV and SPCSV sequences were successfully recovered from the sRNA, but only one full-length viral RNA sequence was recovered. In addition, ssDNA and dsDNA reverse transcribing viruses were identified from the small RNA sequences.

A similar strategy was used to identify viruses present in a *Drosophila* cell line, and from published sRNA datasets for mosquitoes and nematodes [58]. Four viruses (two positive strand ssRNA and two dsRNA viruses) were identified from the *Drosophila* S2-GMR cell line. In addition, two viruses, including one new virus, were identified from the mosquito. However, full length genomes of the viruses could not be assembled from the sRNA datasets. RT-PCR, RACE-PCR and sequencing were used to fill gaps in the viral sequences.

### 4.4. NGS for the Sequencing of Viral Genomes

The traditional approach for the sequencing of viral genomes involves PCR or RT-PCR amplification of DNA or cDNA fragments, and cloning prior to Sanger sequencing for DNA or RNA viruses respectively. For the large DNA viruses such as baculoviruses which have genomes of 80–180 kb, fragments of genomic DNA are generated using restriction enzymes rather than PCR, prior to cloning and sequencing. NGS now provides an alternative approach for the sequencing of large DNA viruses and was used successfully for sequencing of the Glossina pallidipes salivary gland hypertrophy virus (GpSGHV). This virus infects tsetse flies, and has been detrimental to laboratory colonies established for use in a sterile male release program for management of tsetse flies, which transmit the agents of both human and animal trypanosomiasis [61]. GpSGHV is a double-stranded circular DNA virus with a genome of 190 kb with 160 non-overlapping ORFs. The genome sequence was assembled by a combination of (a) shotgun 454-pyrosequencing, (b) Sanger sequencing of a partial genomic cloned library of the viral DNA fragments, and (c) sequence gap filling using PCR products, followed by sequence assembly. NGS data also provided information about the genomic variation of this virus.

NGS was also applied to sequencing of the genome of the polydnavirus, Cotesia vestalis bracovirus (CvBV) [60]. Polydnaviruses (PDV) are associated with parasitoid wasps and serve to suppress the immune system of the parasitized host insect. The genome of PDV is 540 kb and is composed of 35 dsDNA segments. CvBV virions and viral DNA were isolated from the ovaries of 400 to 500 *C. vestalis*. Genome sequencing was performed using 454 GS FLX [76]. Assembled contigs were first compared with the genome of *Cotesia plutellae* bracovirus (CpBV) using blastn, and then validated by PCR. The relationship of the remaining contigs was determined by multiplex PCR [77] and the remaining gaps were sequenced by Sanger sequencing. Sequences were finally assembled using the Phred, Phrap and Consed software programs, and low quality regions of the genome were resequenced. Each circular segment was confirmed by multiplex PCR and sequencing.

### 4.5. Aphid Virus Discovery using Transcriptome and Small RNA Sequencing

We sequenced the soybean aphid transcriptome using Illumina GAII. Total RNA was extracted from aphids using TRIzol reagent (Invitrogen) and transcriptome libraries prepared for single read analysis according to Illumina protocols. Three samples were prepared, one of which had a single polyT purification step, as compared to the others that were subjected to two of these steps. The resulting reads of 75 nt were assembled using Velvet [78] and ABySS [79]. Contigs of >100 nt were screened for viral sequences using blastx and blastn against the NCBI nr database. A novel ssRNA positive-strand virus (named Aphis glycines virus, AGV) that showed homology to tetravirus RdRP (RNA-dependent RNA polymerase) was identified. Two known aphid viruses, Aphid lethal paralysis virus (ALPV) [80] and Rhopalosiphum padi virus (RhPV) [81] were also detected. The contigs that included AGV sequence were used to screen the contig sets by blastn to identify additional contigs with AGV sequences. By using this approach, 95% of the AGV sequence was revealed. In contrast, less than 2% of the genomes of ALPV and RhPV were detected from the transcriptome sequence data, suggesting that these viruses were present at relatively low copy number. The presence of all virus sequences detected in the transcriptome was confirmed in the aphid colony by RT-PCR.

We then used small RNA sequencing using Illumina GAII for detection of RNA viruses in apparently healthy laboratory colonies of the pea aphid (*Acyrthosiphon pisum*), the green peach aphid (*Myzus persicae*) and the soybean aphid (*Aphis glycines*). Total RNA was extracted from aphids using TRIzol and the Illumina Small RNA Sample Prep Kit used for production of sRNA libraries. For the small RNA sequencing reads, 3′-adaptors were removed and the reads were then *de novo* assembled using Velvet [78]. Contigs (>100 nt) were searched for viral sequences by blastx and blastn against the NCBI nr database. Sequences with homology to ALPV were found in the pea aphid and the soybean aphid samples, and a DNA virus, Myzus persicae densovirus (MpDNV) [82,83] was detected in the green peach aphid sample. The pea aphid small RNA reads were also mapped to the full-length Acyrthosiphon pisum virus (APV, unclassified ssRNA positive-strand virus) using a Perl script, but no reads with significant homology to APV were found, suggesting that the aphids did not harbor APV. The soybean aphid small RNA reads were also mapped to AGV, discovered from analysis of the transcriptome sequencing data, and sRNA-derived contigs of AGV were identified.

More than 95% of the ALPV-like genome sequence from *A. pisum* was assembled from siRNA reads into three contigs. Although more than 70% of the AGV genome sequence was covered by the siRNA sequences, none of the contigs generated were more than 300 nt in length. In the case of ALPV from the soybean aphid, and MpDNV from the green peach aphid, less than 30% of the genomes were covered by the assembled siRNA sequences.

## 5. Limitations of NGS for Insect Virus Discovery

Although NGS has been transformative for virus discovery, there are limitations. One limitation of the use of NGS methods is that it is not possible to identify novel viruses that lack homology to known viruses. An exception to this is when the DNA or RNA sequenced is extracted from purified virus, and hence the viral origin of the sequence has already been established. A second limitation to the use of NGS is that full length genome sequences are unlikely to be acquired unless the virus is present in the host insect at high titers. Further sequencing of the genome will likely be required. In some cases, although most of the sequence is acquired, the 5′ and 3′ end sequences are not found [35]. Hence it is important, where possible, to retain frozen tissues for virus isolation and / or maintain a colony of the insect for virus extraction. A third challenge for NGS methods is the use of non-standardized methods for data analysis. There are no clearly established guidelines for acceptable read quality, parameters for short read data assembly, and significance of BLAST hits, for example. With the increasing use of NGS, there is a real need to develop tools and software to handle bioinformatics analysis for any organism, rather than just model organisms.

## 6. Conclusions

Next generation sequencing technologies have fundamentally changed the methodology for discovery of viruses from insects, for diagnostics and epidemiology of viral diseases, and for the study of virus-insect interactions. The sequencing of transcriptomes and small RNAs not only generates viral sequences for assembly into viral genome sequences, but also provides insight into host response to virus infection, the virus-insect interactome. In addition, NGS provides for a quantitative glimpse of both the repertoire of viruses present within an insect, and simultaneous insight into how the host transcriptome responds to virus presence. For example, comparison of the transcriptomes and sRNAs of infected and non-infected populations will indicate how virus infection impacts host gene transcription, and whether the viral RNA is susceptible to degradation by the host RNAi response. The third generation sequencing technology(ies) will likely further improve our ability to discover new insect viruses [84–86].

**Figure 3.**
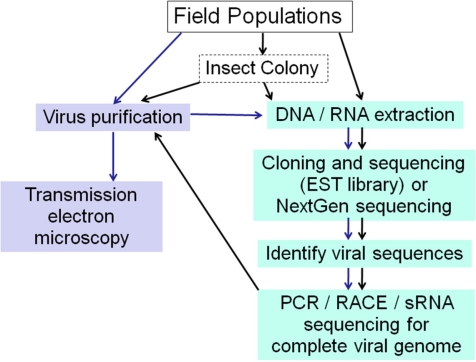
Strategies for Insect Virus Discovery. When viral sequences are discovered in EST libraries or by NGS, frozen material or an insect colony established from field caught specimens is valuable for subsequent virus purification for further analyses.

It is evident from this review that there are multiple approaches for identifying novel insect viruses and for assembly of viral genome sequences (Figure 3). Different methods used for library preparation (total DNA, RNA, small RNA or nucleotides isolated from purified viruses) and the libraries used may result in detection of different viruses.

The examples of novel insect viruses discovered by use of NGS technologies illustrate the ubiquity of viruses in field populations, laboratory colonies of insects and in insect-derived cell lines. These viruses may cause covert or overt infection, or be vectored by the insect to their primary plant or animal hosts. The ubiquity of viruses in laboratory colonies of insects and in cell lines has implications for the use of these tools for analysis of insect-virus interaction. Insects have several anti-viral defense pathways [87], including RNAi [87] and apoptosis [88], and the presence of covert viruses is likely to impact these pathways. Viruses have developed strategies to overcome or suppress host insect anti-viral immunity, by encoding for example, suppressors of RNA silencing [68,89,90] and inhibitors of apoptosis [91]. Hence, as some viruses asymptomatically infect insects, it is possible that RNAi or other insect anti-viral immune pathways are already activated and/or impaired by those viruses. Such a scenario may explain the relative lack of response of the pea aphid to challenge with ALPV [92] for example.

While there is increasing interest in gene silencing approaches for insect pest management, with successful demonstration of the approach for beetles [93], results in other insects including the Lepidoptera have been mixed [94]. It remains to be seen whether the presence of RNA viruses in these insects and their effect on the RNAi processing machinery (inhibition of Argonaute 2 for example [89]), impairs the use of dsRNA for gene silencing for pest management. NGS provides a powerful platform for analysis of pathogens present in test organisms, and the potential interference of covert viruses in experimental outcomes and physiological studies.

## Figures and Tables

**Table 1.** Comparison of the most commonly used next generation sequencing platforms. (Modified from [38]).

**Platform**	**Roche 454/GS FLX +**	**Illumina GAII**		**Life Technologies / SOLiD 5500xl system**
		**GAII**	**HiSeq 2000**	
**Library**	Fragment / emulsion PCR	Fragment / polony		Fragment / emulsion PCR
**Sequencing Principal**	Pyrosequencing	Sequencing by synthesis		Sequencing by ligation
**Read length (base)**	700–1000	150	100	75
**Gb per run**	0.7	95	600	300
**Pros**	Long reads improve mapping in repetitive regions, fast run time	Currently the most widely used platform in the field		Two-base encoding provides inherent error correction
**Cons**	High reagent cost, high error rate in homopolymer repeats	Low multiplexing capability of samples		Long run time
**Examples of biological applications**	Bacterial and insect genome *de novo* assemblies, medium scale (<3 Mb) exome capture, virus discovery in metagenomics	Variant discovery by whole— genome resequencing or whole—exome capture, virus discovery and gene discovery in metagenomics		Variant discovery by whole—genome resequencing or whole— exome capture, gene discovery in metagenomics

**Table 2.** Bioinformatics methods used for virus discovery by Next Generation Sequencing (NGS) data mining [53].

**Sequencing Type**	**DNA**	**Transcriptome**	**Small RNA**
**Sample and library preparation**	Libraries are prepared from DNA isolated from the infected host or from purified viruses.	Libraries are prepared from RNA isolated from the infected host or purified viruses.	Libraries are prepared by isolation of small RNA from host total RNA (∼17–30 nt).
**Treatment of raw data**	Base calling, trim adaptors and remove low quality reads. Cluster reads (optional).		
**Initial BLAST analysis & assembly**	BLAST analysis/mapping followed by assembly of the reads that have significant hits to viral sequences; or assembly of reads and BLAST analysis of the resulting assembled contigs.		Assemble reads followed by BLAST analysis/mapping.
**Isolating potential virus sequences**	Separate contigs with significant hits (e-value: ≤ 1 × 10^−3^) to viruses from non-virus hits.		
**Re-assemble to generate longer virus contigs**	Re-assemble the contigs that hit viral sequences by using various assembly programs (for example software used for Sanger sequence assembly) to generate longer contigs.		
**BLAST analysis to identify viruses (known and novel)**	BLAST the assembled contigs against non-redundant (nr) databases and virus databases.		
**Extend virus genome with overlapping reads with little sequence similarity to known viruses**	Identify contigs with hits to viruses [e-value: ≤ 1 × 10^−5^]. BLAST the viral contigs against the total contig set to search for contigs that overlap viral contigs (but were not identified by BLAST against nr or viral databases). This step is important for identification of novel viral sequences. Assemble virus genomes.		
**Generate complete virus genome**	Fill the sequence gaps by PCR (RT-PCR, RACE-PCR) and Sanger sequencing.		
**Characterize virus**	Further characterization of virus (classification, localization, transmission, host range). Refer to polythetic criteria for virus group for parameters needed to facilitate virus classification [54].		

**Table 3.** Insect viruses detected/discovered by use of Next Generation Sequencing technologies.

**Virus**	**Origin**	**Reference**
Birnaviridae (dsRNA)		
Drosophila X virus (DXV)	*D. melanogaster* cell line (S2-GMR)	[58]
Drosophila birnavirus (DBV)*	*D. melanogaster* cell line (S2-GMR)	[58]
Totiviridae (dsRNA)		
Drosophila totivirus (DTV)*	*D. melanogaster* cell line (S2-GMR)	[58]
Dicistroviridae (+ssRNA)		
Drosophila C virus (DCV)	*D. melanogaster* ovary somatic cell line	[58]
Black queen cell virus (BQCV)	*Apis mellifera*	[41]
Kashmir bee virus (KBV)	*Apis mellifera*	[41]
Acute bee paralysis virus (ABPV)	*Apis mellifera*	[41]
Isreali acute paralysis virus (IAPV)	*Apis mellifera*	[41,57]
Aphid lethal paralysis virus-AP (ALPV-AP)	*Acyrthosiphon pisum*	[59]
ALPV-AG	*Aphis glycines*	[59]
ALPV-Brookings strain (ALPV-Brookings)*	*Apis mellifera*	[35]
Big Sioux river virus (BSRV)*	*Apis mellifera*	[35]
Nodaviridae (+ssRNA)		
American nodavirus (ANV)*	*D. melanogaster* cell line (S2-GMR)	[58]
Mosquito nodavirus (MNV)*	*Aedes aegypti*-Liverpool strain	[58]
Nidovirales (+ssRNA)		
Cavally virus (CAVV)*	Mosquito heads (multiple species)	[17]
Tetraviridae (+ssRNA)		
Drosophila tetravirus (DTrV)*^1^	*D. melanogaster* cell lines, S2-GMR & Kc	[58]
Togaviridae (+ssRNA)		
Sindbis virus (SINV)	*Aedes aegypti*-Liverpool strain	[58]
Picornaviridae (+ssRNA)		
Deformed wing virus (DWV)	*Apis mellifera*	[41]
Sacbrood virus (SBV)	*Apis mellifera*	[41]
Polydnaviridae		
Costesia vestalis bracovirus (CvBV)	*Costesia vestalis*	[60]
Parvoviridae (ssDNA)		
Myzus persicae densovirus (MpDNV)	*Myzus persicae*	[59]
Unclassified		
Noravirus (+ssRNA)*	*D. melanogaster* ovary cell line	[58]
Chronic bee paralysis virus (CBPV; +ssRNA)	*Apis mellifera*	[41]
Glossina pallidipes salivary gland hypertrophy virus (GpSGHV;dsDNA)	*Glossina pallidipes* salivary glands	[61]
Lake Sinai Virus 1 (LSV1;+ssRNA)*	*Apis mellifera*	[35]
Lake Sinai Virus 2 (LSV2;+ssRNA)*	*Apis mellifera*	[35]
Aphis glycines virus (AGV;+ssRNA)*	*Aphis glycines*	[59]
Others		
Many DNA viruses (known and novel) from animal, plant, insect	Various species of female mosquitoes	[42]
Many known DNA and RNA viruses	*Apis mellifera*	[35]

* indicates novel viruses;

1 Based on the sequence, DTrV is actually Drosophila A virus.
